# MR-Based Morphometry of the Posterior Fossa in Fetuses with Neural Tube Defects of the Spine

**DOI:** 10.1371/journal.pone.0112585

**Published:** 2014-11-13

**Authors:** Ramona Woitek, Anton Dvorak, Michael Weber, Rainer Seidl, Dieter Bettelheim, Veronika Schöpf, Gabriele Amann, Peter C. Brugger, Julia Furtner, Ulrika Asenbaum, Daniela Prayer, Gregor Kasprian

**Affiliations:** 1 Department of Biomedical Imaging and Image-guided Therapy, Medical University of Vienna, Vienna, Austria; 2 Public Hospital Wiener Neustadt, Wiener Neustadt, Austria; 3 Department of Paediatrics and Adolescent Medicine, Medical University of Vienna, Vienna, Austria; 4 Department of Obstetrics and Gynecology, Medical University of Vienna, Vienna, Austria; 5 Department of Clinical Pathology, Medical University of Vienna, Vienna, Austria; 6 Center for Anatomy and Cell Biology, Medical University of Vienna, Vienna, Austria; The University of Kansas Medical Center, United States of America

## Abstract

**Objectives:**

In cases of “spina bifida,” a detailed prenatal imaging assessment of the exact morphology of neural tube defects (NTD) is often limited. Due to the diverse clinical prognosis and prenatal treatment options, imaging parameters that support the prenatal differentiation between open and closed neural tube defects (ONTDs and CNTDs) are required. This fetal MR study aims to evaluate the clivus-supraocciput angle (CSA) and the maximum transverse diameter of the posterior fossa (TDPF) as morphometric parameters to aid in the reliable diagnosis of either ONTDs or CNTDs.

**Methods:**

The TDPF and the CSA of 238 fetuses (20–37 GW, mean: 28.36 GW) with a normal central nervous system, 44 with ONTDS, and 13 with CNTDs (18–37 GW, mean: 24.3 GW) were retrospectively measured using T2-weighted 1.5 Tesla MR -sequences.

**Results:**

Normal fetuses showed a significant increase in the TDPF (r = .956; p<.001) and CSA (r = .714; p<.001) with gestational age. In ONTDs the CSA was significantly smaller (p<.001) than in normal controls and CNTDs, whereas in CNTDs the CSA was not significantly smaller than in controls (p = .160). In both ONTDs and in CNTDs the TDPF was significantly different from controls (p<.001).

**Conclusions:**

The skull base morphology in fetuses with ONTDs differs significantly from cases with CNTDs and normal controls. This is the first study to show that the CSA changes during gestation and that it is a reliable imaging biomarker to distinguish between ONTDs and CNTDs, independent of the morphology of the spinal defect.

## Introduction

The prenatal differentiation between open (ONTDs) and closed neural tube defects (CNTDs) of the spine is crucial because postnatal prognoses differ considerably. The differentiation based on MRI findings of the neural tube defect (NTD) can be impeded by limited depiction and distinction of the anatomical structures at the site of the NTD, especially in early second-trimester fetuses due to limited spatial resolution. As a generally accepted rule, ONTDs are almost always associated with Chiari II malformations, except for very few cases where CNTDs have been reported in association with Chiari II malformations [1]–[3]. In a fetus with Chiari II malformation the posterior fossa is small due to hypoplasia of the supraoccipital, exoccipital and basioccipital parts of the occipital bone resulting in caudal displacement of the vermis cerebelli and the brainstem. Associated supratentorial abnormalities are dysgenesis of the corpus callosum, enlarged massa intermedia, hydrocephalic lateral ventricles, polymicrogyria, tectal beaking, hemispheric interdigitations and cortical heterotopias, and the so-called Luckenschaedel or lacunar skull [4]–[6]. Currently, various imaging signs of a morphologically abnormal posterior fossa have been described by ultrasound. These non-quantitative criteria comprise the banana sign [7]–[9], an effaced cisterna magna [10], bilateral downward-triangle shape (triangle sign), quadrilateral angular shape (square sign) of the lateral ventricles, [11] and absence of the translucency of the fourth ventricle [12].

Only few studies exist that report quantifiable criteria on prenatal ultrasound or MRI in large numbers of fetuses: the clivus-supraocciput angle (CSA) (evaluated on ultrasound in normally developing fetuses and in fetuses with Chiari II malformations) [10], [13], calculation of the posterior fossa volume (MRI-based, investigated in normally developing fetuses only) [14], the sagittal diameter of the brainstem, the brainstem to occipital bone diameter, and the ratio of the first to the latter (examined in normally developing fetuses and in fetuses with Chiari II malformations on ultrasound) [15].

This fetal MR study aims to evaluate the formerly described CSA [10], [13] and the maximum transverse diameter of the posterior fossa (TDPF) as morphometric parameters to aid in the reliable diagnosis of either ONTDs or CNTDs. The two parameters CSA and TDPF were chosen as they can both be easily measured by prenatal ultrasound or MRI.

## Methods

The Ethics Committee of the Medical University of Vienna approved the protocol for this study and we conducted the study according to the Declaration of Helsinki. Written informed consent was waived by the ethics committee due to the retrospective study design.

### Patients

All 289 patients were referred to our department for fetal MRI between 2006 and 2014 to rule out or to confirm suspicious findings on fetal ultrasound. The established gestational age was based on ultrasound examinations during the first trimester. MRI revealed no pathology or isolated congenital pathologies that did not affect the CNS (n = 238). The most common pathologies were congenital diaphragmatic hernia, gastroschisis, cleft lip and palate, esophageal atresia, and cystic adenomatous malformation of the lung. Patient data were anonymized and de-identified prior to analysis.

### MRI

MRI examinations were performed on a 1.5 Tesla system (Philips Medical Systems, Best, The Netherlands) with a five-element, phased-array cardiac coil. No contrast agents or sedation were used.

The MRI protocol included axial, and coronal T2-weighted single-shot fast spin-echo (SSFSE) sequences, and/or steady-state free precession (SSFP) sequences. All sequences were acquired as previously proposed [16] and adjusted according to the changing structural composition of the fetal brain during gestation.

### Image Evaluation

**Figure 1:**
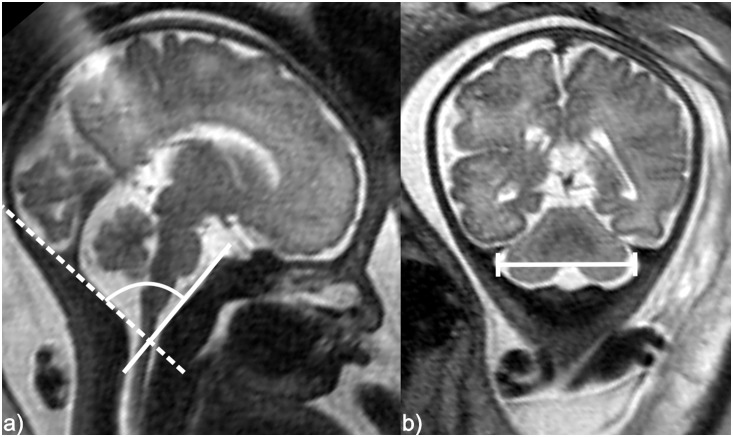
Schematic drawings of the CSA and the TDPF a) Measurement of the CSA. Midsagittal T2-weighted SSFSE MR image of a fetus at 33 GW. Continuous line along the postero-superior surface of the clivus connecting the most cranial part of the clivus with the anterior border of the foramen magnum (basion). Dashed line along the antero-superior surface of the supraocciput cutting the posterior border of the foramen magnum (opisthon). The angle between these two lines is the CSA. b) Measurement of the TDPF. Coronal T2-weighted MR image of a fetus at 33 GW. The distance between the medial surfaces of the lateral bony margins of the posterior fossa at the level of the lateral insertions of the tentorium cerebelli was measured.

Image evaluation was performed separately for each fetus by one reader, in fetuses with NTDs (resident with experience in fetal MRI and in neuroimaging) and in the cohort of 238 fetuses with a normal CNS (medical student extensively trained by a professor of radiology with the subspecialties of neuroradiology and fetal MRI). A sample of 75 fetuses with normal CNS was evaluated by both readers blinded for results not obtained by themselves. On midsagittal slices of T2-weighted SSFSE or SSFP MR sequences, the CSA was measured using two lines: The first line was placed along the postero-superior surface of the clivus, connecting the most cranial part of the clivus and the anterior border of the foramen magnum (basion) (continuous line in Figure 1a). The second line was placed along the superior surface of the supraocciput, cutting the posterior border of the foramen magnum (opisthon) (dashed line in Figure 1a). These anatomical structures could be easily identified, as there is high contrast between the hypointense bony clivus and supraocciput and the adjacent hyperintense cerebrospinal fluid (CSF) on these sequences. The angle at which the continuous and the dashed lines intersected was measured as the CSA. The angle was measured on up to three midsagittal images, each from a different MR sequence, depending on the number of acquired sagittal T2-weighted SSFSE or SSFP sequences. Consequently, the mean value of all measurements was used for statistical evaluations.

To measure the distance between the medial surfaces of the lateral bony margins of the posterior fossa at the level of the lateral insertions of the tentorium cerebelli (Figure 1b), the largest distance between the lateral borders of the posterior cranial fossa at the level of the insertion of the tentorium cerebelli was depicted (TDPF) on coronal T2-weighted SSFSE or SSFP sequences. In each fetus, the distance was measured on up to three images, each from a different MR sequence, depending on the number of acquired MR sequences. The mean value of all measurements in one fetus was then used for statistical evaluations.

**Figure 2:**
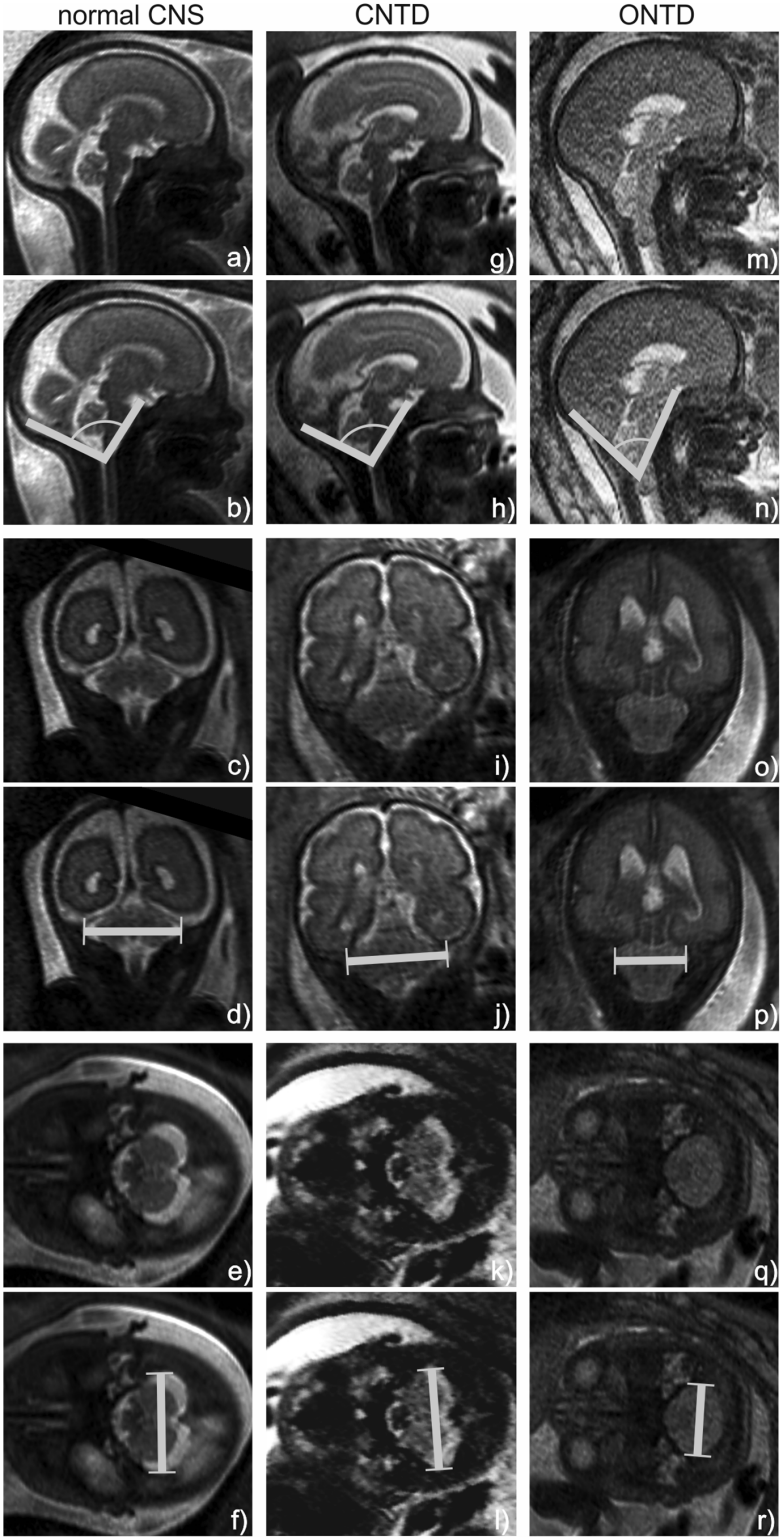
Line drawings show measurements of the CSA and TDPF performed on MR images of three exemplar fetuses: one fetus with normal CNS development at 27 GW (a–f), one fetus with a CNTD at 28GW (g–l) and one fetus with an ONTD at 27 GW (m–r). The CSA was measured on midsagittal T2-weighted SSFSE MR images (a+b, g+h, m+n). The TDPF was measured on coronal T2-weighted MR images (c+d, i+j, o+p) or on axial T2-weighted MR images (e+f, k+l, q+r).

Three fetuses at similar gestational ages are shown in Figure 2 in order to illustrate the way the measurements of the CSA and TDPF were performed in fetuses with normal CNS development, with an ONTD or CNTD.

### Inclusion criteria

Only fetuses with unequivocal imaging results with regard to the presence of either an ONTD or CNTD were included in our study.

### Exclusion criteria

MR sequences with severe fetal head movement were excluded. If the CSA or the TDPF could not be measured on midsagittal or coronal images, respectively, the entire sequence was excluded from analysis. Fetuses of multiple pregnancies, with intrauterine growth restriction, or with neural pathologies other than those related to neural tube defects of the spine, were excluded.

### Statistical Evaluation

Statistical planning and analysis were performed by a statistician (M.W.) using the SPSS 17.0 software package for Microsoft Windows, SPSS, Chicago, Ill). The study cohort was divided into three groups: fetuses with ONTDs of the spine; fetuses with CNTDs of the spine; and fetuses with normal CNS development. For each of those fetuses with NTDs of the spine, an age-matched control fetus was selected from the group of fetuses with normal neural development, with a maximum difference of +/−3 days of gestation. This age-based matching was performed because both the CSA and the TDPF increased with gestational age. To estimate interrater reliability the differences between the measurements performed by the two readers in a sample of 75 fetuses with normal CNS were calculated for the CSA and DTPF. Pearson correlation coefficients were computed to estimate consistency between the two readers.

The minimum and maximum values for the clivus-supraocciput angle and the TDPF were assessed, and mean values were calculated, as well as standard deviations and the 10^th^ and 90^th^ percentiles for both measurements. Pearson correlation was used to calculate correlations of gestational age with the CSA or TDPF in fetuses with normal CNS development and in fetuses with NTDs. A mixed model analysis of variance and student’s t-tests for dependent samples were used to calculate differences between fetuses with normal CNS development and fetuses with ONTDs or CNTDs, respectively. Student’s t-tests for independent samples were used to calculate differences between fetuses with ONTDs and with CNTDs. A p-value equal to or below 0.05 was considered statistically significant.

## Results

Between 2006 and 2014 a NTD has been diagnosed in 65 fetuses based on MRI at our institution. The diagnoses could be confirmed in 44 cases of ONTDs and in 13 cases of CNTDs by postnatal surgery or postmortem examination. In 6 fetuses no follow up information was available, therefore they were excluded from our study.

Terminations of pregnancy were performed in 24 fetuses with ONTDs between 18 and 35 GW based on the diagnoses established using fetal ultrasound and MRI and on maternal α-fetoprotein levels. Due to the retrospective study design no measurements comparable to our MR-based morphometry were performed during postmortem examination. 33 fetuses with NTDs were born alive after cesarian section.

Two fetuses with ONTDs had to be entirely excluded due to severe head motion and insufficient overall image quality in one case, and due to twin pregnancy in another case. In one ONTD the CSA was not reliably measurable retrospectively and in three ONTDs the DMPF was not reliably measurable retrospectively, all due to motion artifacts on those sequences necessary to perform measurement. Descriptive statistics for the subgroups of included fetuses with ONTDs (n = 44), CNTDs (n = 13), and normal CNS development (n = 238) are shown in Table 1.

**Table 1:** Descriptive statistics of groups of fetuses with ONTDs and CNTDs and their respective control fetuses with normal CNS development.

		gestational age [weeks]			
	n	mean ± std dev	min–max	clivus-supraocciputangle [°]	max. DM of theposterior fossa [mm]
ONTDs	44	24.7±5.1	17–37	53.4±10.4	22.4±5.8
controls	44	24.7±5.2	17–37	78.0±8.5	32.3±9.0
CNTDs	13	26.2±2	19–33	75.0±11.1	25.1±6.5
controls	13	26.3±5.3	19–33	80.0±8.8	37.2±8.7

n = number of fetuses, std dev = standard deviation, min = minimum, max = maximum, DM = diameter.

**Figure 3:**
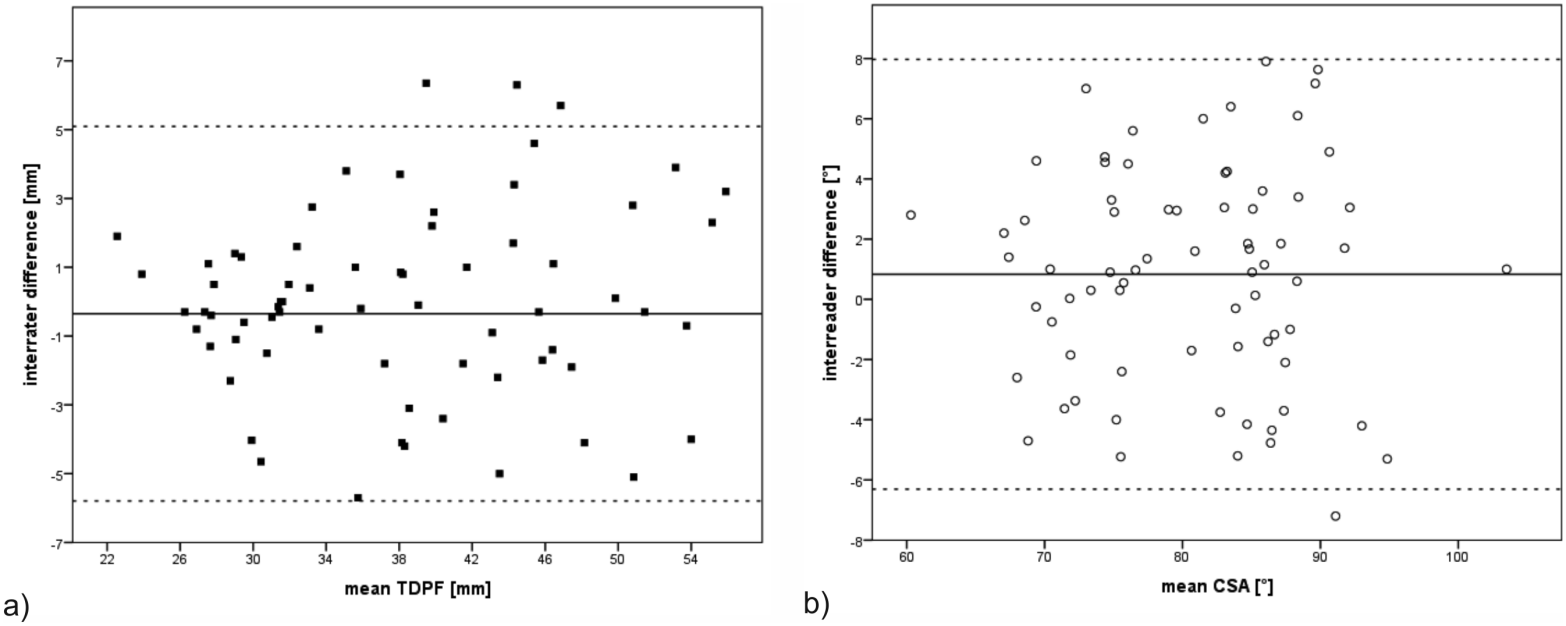
Measurements of the TDPF and CSA by two readers. a) Mean TDPF measured by the two readers plotted against the interrater difference. Continuous line represents overall mean difference (0.4 mm). Dashed lines represent 95% confidence intervals. b) Mean CSA measured by the two readers plotted against the interrater difference. Continuous line represents overall mean difference (0.8°). Dashed lines represent 95% confidence intervals.

In an exemplar subgroup of 75 fetuses with normal CNS TDPF and CSA were measured by two readers (R.W., A.D.) and assessed for interrater reliability (Figure 3; Table 2). Correlations between the two readers with regards to the CSA and the TDPF were high (r = .95; p<.001).

**Table 2:** Comparison between the two readers regarding CSA and TDPF.

	Reader 1		Reader 2		mean difference	range of differences	r	sig
	mean	std dev	mean	std dev				
CSA [°]	81.0	8.2	80.0	8.4	1.0	0–8.2	0.95	<.001
TDPF [mm]	38.1	8.5	38.1	8.7	0.0	0–6.4	0.95	<.001

Std dev = standard deviation, sig = level of significance.

**Figure 4:**
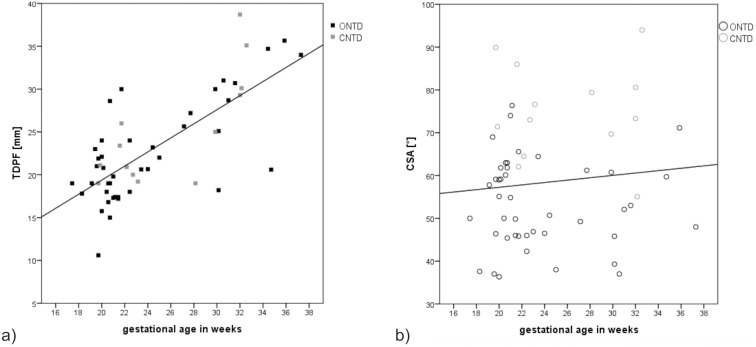
The TDPF and CSA in fetuses with ONTDs and CNTDs plotted against gestational age. a) Correlation of the TDPF with gestational age was significant (r = .943, p<.000) (see correlation line). b) The CSA in fetuses with ONTDs and CNTDs plotted against gestational age. Correlation was low and insignificant (r = .103, p = .452).

**Figure 5:**
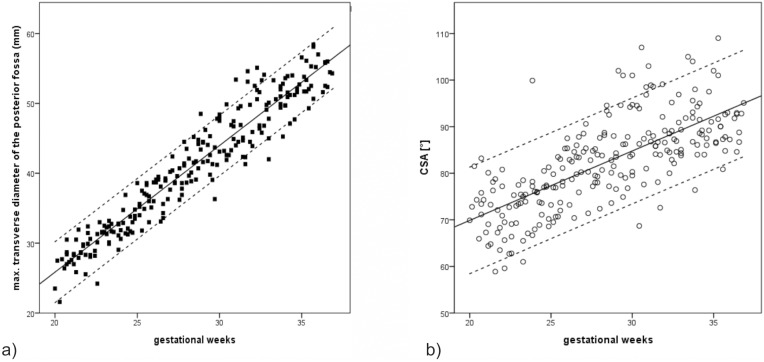
Correlations of the TDPF and CSA with gestational age. a) Correlation between the TDPF and gestational age in 238 fetuses with normal CNS development. Squares show TDPF values. The regression line is continuous (r = .956; p<.001). Dashed lines show calculated 10th and 90th percentiles. b) Correlation between the CSA and gestational age. Circles show CSA values of 238 fetuses with normal CNS development. The regression line is continuous (r = .714; p<.001). Dashed lines show 10th and 90th percentiles.

When we began evaluating fetuses with NTDs (17–37 GW, mean: 24.3 GW), we observed a significant correlation between gestational age and the TDPF (r = .943, p<.000) (Figure 4a), and a lower and insignificant correlation between the CSA and gestational age (r = .103, p = .452) (Figure 4b). As the finding concerning the CSA was in contrast to previously published results [10], we subsequently evaluated 238 fetuses with normal CNS development to obtain their CSA and TDPF (20–37 GW, mean: 28.36 GW) to confirm whether there was an increase in the CSA during gestation. These 238 fetuses were homogeneously distributed with regard to their gestational age, with 13–15 fetuses in each gestational week except for the 21^st^ and 37^th^ gestational weeks, in which there were only 10 and nine fetuses, respectively. Mean values, standard deviations, 10^th^ and 90^th^ percentiles for each gestational week between the 21^st^ and 37^th^ gestational week, and mean and standard deviations for all fetuses with a normal CNS development are shown in Table 3. In this cohort of fetuses, we found a significant and very high correlation between gestational age and the TDPF (r = .956; p<.001) (Figure 5a), and a significant correlation between gestational age and the CSA (r = .714; p<.001) (Figure 5b).

**Table 3:** Descriptive statistics of fetuses with normal CNS development, ONTDs and CNTDs.

	TDPF [mm]										CSA [°]										
GW	normal				ONTDs			CNTDs			normal				ONTDs			CNTDs			GW
	mean	std dev	0.1	0.9	mean	max	min	mean	max	min	mean	std dev	0.1	0.9	mean	max	min	mean	max	min	
21	26.9	2.6	23.6	30.2	18.0	22.0	16.0				74.2	5.1	67.7	80.8	54.7	63.0	36.3				21
22	28.4	1.7	26.2	30.6	18.0	20.0	17.0	26.0	29.0	23.0	70.2	6.5	61.9	78.4	54.1	74.0	45.9	76.4	79.4	73.3	22
23	30.0	2.3	27.0	32.9	21.0	21.0	21.0	21.0	21.0	21.0	69.6	5.9	62.0	77.3	44.3	44.3	44.3	71.4	71.4	71.4	23
24	31.7	1.2	30.1	33.2	22.0	22.0	21.0	19.0	19.0	19.0	74.6	8.8	63.3	85.8	54.7	64.5	46.9	59.0	59.0	59.0	24
25	32.9	1.7	30.7	35.1	22.0	23.0	21.0				74.4	4.2	68.9	79.8	48.6	50.7	46.5				25
26	36.0	2.0	33.4	38.5							76.8	5.1	70.3	83.3							26
27	37.3	2.3	34.4	40.2							81.5	5.1	74.9	88.0							27
28	39.7	2.2	36.8	42.5	26.0	27.0	26.0				82.0	4.9	75.7	88.3	51.1	50.7	49.3				28
29	42.1	2.8	38.5	45.7				19.0	19.0	19.0	84.3	6.9	75.5	93.2				86.0	86.0	86.0	29
30	42.6	3.0	38.8	46.4	30.0	30.0	30.0				86.0	8.6	75.1	97.0	60.8	60.8	60.8	89.9	89.9	89.9	30
31	45.6	2.6	42.3	49.0	25.0	30.0	18.0				88.8	10.4	75.4	102.1	47.8	57.4	39.3				31
32	47.0	4.3	41.5	52.6	30.0	31.0	29.0				88.8	8.3	78.2	99.5	52.6	53.0	52.1				32
33	49.6	3.5	45.1	54.0	34.0	34.0	34.0	33.0	39.0	29.0	87.3	4.6	81.4	93.2	65.1	65.1	65.1	65.6	94.0	45.1	33
34	49.4	3.4	45.0	53.8							91.2	8.2	80.7	101.7							34
35	51.2	2.1	48.5	53.9	28.0	35.0	21.0				89.4	4.1	84.3	94.7	63.6	67.4	59.7				35
36	53.9	2.8	50.3	57.5	36.0	36.0	36.0				91.7	7.6	81.9	101.4	71.2	71.2	71.2				36
37	54.4	1.9	52.0	56.9							90.3	3.6	85.7	94.9							37
**all**	**41.1**	**9.1**									**82.4**	**7.6**									**all**

GW = gestational week, TDPF = maximum transverse diameter of the posterior fossa, CSA = clivus-supraocciput angle, ONTD = open neural tube defect, CNTD = closed neural tube defect, std dev = standard deviation, 0.1 = 10^th^ percentile, 0.9 = 90^th^ percentile; max = maximum, min = minimum.

**Figure 6:**
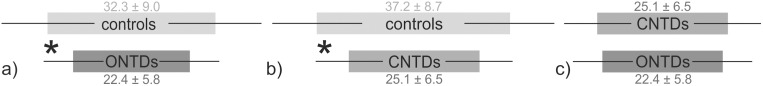
Comparisons of the TDPF in ONTDs, CNTDs and the respective control groups. Bars represent mean values and lines represent standard deviations of the TDPF in fetuses with ONTDs (a, c), in fetuses with CNTDs (b, c) and their respective age matched control fetuses with normal CNS development (a, b). Asterisks indicate significant differences between ONTDs and control fetuses with normal CNS and between CNTDs and control fetuses with normal CNS development (p<.001).

**Figure 7:**
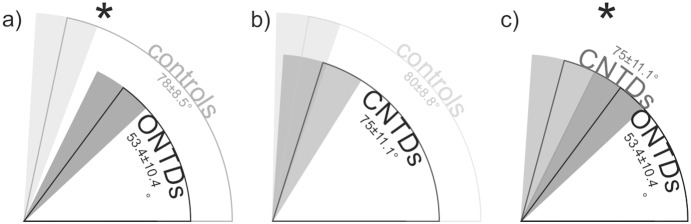
Comparisons of the CSA in ONTDs, CNTDs and the respective control groups. Sector outlines represent mean values and gray sectors represent standard deviations of the CSA in fetuses with ONTDs (a, c), with CNTDs (b, c) and their respective age matched control fetuses with normal CNS development (a, b). Asterisks indicate significant differences between ONTDs and control fetuses with normal CNS and between ONTDs and CNTDs (p<.001).

In both groups of fetuses with ONTDs and CNTDs, the TDPF was significantly different from their age-matched control fetuses (p<.001 for both groups) (Figure 6). The TDPF was not significantly different when directly comparing fetuses with ONTDs to fetuses with CNTDs of the spine (p = .677) (Figure 7).

Only in fetuses with ONTDs was the CSA significantly different from their age-matched control fetuses (p<.001), whereas, in CNTDs, it was not (p = .163). The CSA was also significantly different when comparing fetuses with ONTDs to those with CNTDs of the spine (p<.001) (Figure 7). Gestational age did not significantly differ in groups of ONTDs and CNTDs (p = .623).

## Discussion

In this study, a comparably large number of normal fetuses and fetuses with ONTDs and CNTDs were evaluated with regard to their CSA and TDPF in order to be able to differentiate between the two entities based on posterior fossa morphometry.

In contrast to d’Addario et al. [10], we were able to prove that, based on the results of the present and a recent study by Grant et al. [17], there is a low but significant correlation of the CSA with gestational age both in fetuses with NTDs and in fetuses with a normal CNS. Thus, our data represent the most extensive analysis of the CSA and TDPF to date, as Grant et al. [17] included a smaller number of fetuses. Increasing values of both the CSA and TDPF during gestation imply that normal values (mean, standard deviation, and 10th and 90^th^ percentiles) have to be established for each gestational week based on a sufficient number of measurements.

In both our study and the study by Grant et al., measurements of the CSA were performed on MR images [17], whereas the measurements that remained constant during gestation obtained by d’Addario et al. were based on ultrasound examinations [10]. The difference between the ultrasound data and the present data may be related to methodological differences. T2-weighted SSFSE and SSFP MR-sequences are free of sonographic shadowing by osseous structures, and offer a very high contrast between the hypointense bony structures (clivus, supraocciput) and the CSF spaces of the posterior fossa. The lines by which the CSA is measured are positioned at the interfaces between these high and low signal intensities. Therefore, MRI might serve as a better tool than ultrasound to precisely identify these structures.

Aside from these differences, the mean values obtained by d’Addario et al. and Grant et al. are comparable to our findings. D’Addario et al. reported the mean value of the CSA in normal fetuses to be 79.3° which is within the 10^th^ and 90^th^ percentile of our measurements in the majority of fetuses (GW 21 and 24–32) [10]. Grant et al. measured a mean CSA of 75.4° in normally developing fetuses [17], while, in our study, the mean CSA of all fetuses with a normal CNS was 82.4°. Grant et al. reported that, in fetuses with Chiari II malformations, between 19 and 25 GW the mean CSA was 57.8°, which is comparable to our mean CSA in fetuses with ONTDs of 53.4°.

A group-wise comparison of fetuses with ONTDs and CNTDs revealed a significant difference with regard to the CSA but not the TDPF. Differentiation between ONTDs and CNTDs is limited, based on the TDPF, as standard deviations of the TDPF overlap in all three comparisons (ONTDs versus controls, CNTDs versus controls, and ONTDS versus CNTDs), with the highest overlap in the comparison of ONTDs to CNTDs. In contrast, the standard deviations calculated for the CSA in ONTDs and controls do not overlap. Comparisons of the CSA in CNTDs versus age-matched controls, or CNTDs versus ONTDs, revealed overlapping standard deviations. With regard to CNTDs versus ONTDs, in particular, this overlap is small and could, theoretically, be overcome with a higher number of measurements. Supported by our data we believe the CSA to be a sensitive tool to distinguish between ONTDS and CNTDs on fetal MRI.

Our findings reveal a smaller TDPF in fetuses with CNTDs than with a normal CNS development, implying that, in CNTDs, the posterior fossa also undergoes malformation, but apparently to a lesser extent than in ONTDs. The hydrodynamic theory [18]–[20] cannot explain this observation. More probably, a genetic background might be the missing link between the pathogenesis of ONTDs and CNTDs and might explain the different development of the posterior fossa.

It has been shown that members of the HOX gene family play important roles both in the formation of the neural tube [21] and in the growth of the enchondral bones of the skull base [22], eventually contributing to the formation of NTDs and Chiari malformations [23]. Most studies on the genetic background of NTDs focus on ONTDs or evaluate pooled data from NTDs without classifying according to ONTDs and CNTDs, mostly due to the small numbers of patients and the lack of patients with CNTDs. Therefore, a study aimed at the distinction of genetic factors that contribute to the formation of ONTDs and CNTDs should be undertaken. A multicentric approach appears to be necessary to be able to include large enough numbers of patients with ONTDs or CNTDs.

The limitations of our study are, firstly, that the evaluation of fetuses was limited to fetuses aged 17–37 GW. Secondly in those fetuses with NTDs that were lost to follow up differentiation between ONTDs and CNTDs was based on imaging findings alone. Furthermore, as this was a single center study, the number of fetuses with NTDs was limited to those patients referred to our MRI unit for diagnostic work-up.

This study shows that the skull base morphology in fetuses with ONTDs differs significantly from cases with CNTDs and normal controls. We show for the first time that the CSA is a reliable imaging biomarker to distinguish between ONTDs and CNTDs in early pregnancy, regardless of the morphology of the spinal defect. Furthermore, our work raises questions concerning the pathogenesis of Chiari II malformations in relation to ONTDs, and also, CNTDs, possibly initiating future studies that could focus on a genetic basis as a link between these two entities.
